# Systematic Review of Complementary and Alternative Veterinary Medicine in Sport and Companion Animals: Therapeutic Ultrasound

**DOI:** 10.3390/ani12223144

**Published:** 2022-11-14

**Authors:** Anna Boström, Kjell Asplund, Anna Bergh, Heli Hyytiäinen

**Affiliations:** 1 Department of Equine and Small Animal Medicine, Faculty of Veterinary Medicine, University of Helsinki, P.O. Box 57, 00014 Helsinki, Finland; 2 Department of Public Health and Clinical Medicine, Umeå University, SE 901 87 Umeå, Sweden; 3 Department of Clinical Sciences, Swedish University of Agricultural Sciences, SE 750 07 Uppsala, Sweden

**Keywords:** therapeutic ultrasound, veterinary medicine, complementary and alternative veterinary medicine, companion animal, sports animal, dog, horse, donkey, cat, musculosketetal disorder, bone healing, contraception

## Abstract

**Simple Summary:**

Therapeutic ultrasound (TU) is used in sport and companion animals to treat diseases and injuries affecting tendons, ligaments, muscles, joints, and bones. Usually, there are 2–6 treatment sessions weekly for up to 4 weeks. The scientific evidence for the treatment has been questioned. We have therefore performed a systemic review of the scientific literature on TU used in dogs, horses, donkeys, and cats. The review shows that there is insufficient scientific evidence for favourable effects in conditions affecting tendons, ligaments, muscles, and joints in these species. The studies have been few and most of them involve only a small number of animals. Many studies also have methodological problems with compromised study quality. When beneficial results are reported, they have not been repeated in independent studies. Favourable effects on bone healing have, however, been reported in experiments where bone fractures have been created surgically in dogs. There is also scientific evidence that TU treatment of testicles in dogs and cats arrests the production of sperm, indicating that it may be used for contraception. The favourable effects on bone healing and the conceptive effects need to be confirmed in high-quality clinical trials.

**Abstract:**

Background: To explore the scientific evidence for therapeutic ultrasound (TU), we conducted a systematic review of the literature on TU in dogs, horses, donkeys, and cats. Methods: In three major databases, relevant articles published in 1980–2020 were identified. The risk of bias in each article was evaluated. Results: Twenty-four relevant articles on the effects of TU in dogs, nine in horses, two in donkeys, and one in cats were identified. TU usually involved 2–6 treatments weekly for up to 4 weeks. Articles on tendon, ligament, and bone healing, acute aseptic arthritis, osteoarthritis, paraparesis, hindquarter weakness, and back muscle pain were identified. In experimental bone lesions in dogs, there is moderate scientific evidence for enhanced healing. For the treatment of other musculoskeletal conditions, the scientific evidence is insufficient due to the high risk of bias. There is substantial evidence that continuous TU increases tissue temperature in muscles and tendons by up to 5 °C in healthy animals. For disorders in tendons, ligaments, muscles, and joints in sport and companion animals, there is insufficient evidence for the clinical effects of TU.

## 1. Introduction

Therapeutic ultrasound (TU) is widely used by veterinarians, animal physiotherapists, and complementary and alternative practitioners to treat musculoskeletal disorders in sport and companion animals [1]. Typically, the treatment consists of daily sessions or 2–3 sessions per week over 3–4 weeks. The therapy may be given in continuous or pulsed mode, and the frequency used may be low (e.g., around 40 kHz) or high (up to 3.5 MHz). The intensity (surface power density) used is typically 1.0–1.5 W/cm^2^ [1], however, doses of 0.2 W/cm^2^ may also be used.

Tissue absorption of soundwaves causes thermal effects, with increased tissue temperature and blood flow. Non-thermal effects associated with TU include acoustic (micro)streaming of fluids [2] and the formation of small vapour-filled cavities in tissue fluids [3]. Observations also suggest that the absorption of mechanical energy may modify gene expression, growth factors, and collagens [4]. Together, thermal and non-thermal effects on the target tissue have been proposed to result in increased local metabolism, circulation, extensibility of connective tissue, and tissue regeneration [5,6].

In human clinical practice, TU was generally introduced in the 1950s. However, systematic reviews have failed to find evidence from high-quality or intermediate-quality studies that the treatment is superior to a placebo in promoting fracture healing [7] or, in early reviews, in treating various other conditions affecting muscles, tendons, ligaments, and joints [8,9].

In animals, the scientific support for the beneficial effects of TU has also been queried. To date, no systematic review on this subject has been published. The present systematic review is one in a series of systematic reviews in this special issue of Animals on methods used in complementary and alternative practice to treat sport and companion animals. The aim was to assess the scientific evidence for the therapeutic use of ultrasound in dogs, cats, horses, and donkeys—regardless of indication. We included both interventional and observational studies. The literature on extracorporeal shock wave therapy, which may be considered to be a special variant of TU using lower frequency sound waves [10], is the subject of a separate review in this special issue of Animals.

## 2. Materials and Methods

In August 2020, professional librarians searched the literature in the databases of Web of Science Core Collection, CABI (Center for Agriculture and Bioscience International), and PubMed (1980–2020). The basic literature search terms, common to all reviews in this review series, were dog OR cat OR horse, AND veterinary medicine OR veterinarian, AND therapy* OR treatment* [11]. For the present review, the additional specific search terms were ultrasound therapy OR therapeutic ultrasound. Two authors [HH, ABo] performed the selection and review of articles. To identify relevant articles missed in the database literature search, reference lists of included articles were scrutinized (“snowballing”). For included articles, we also reviewed articles in Google Scholar that cited the articles we had already identified to find any additional relevant articles that might have been missed by other search modes. Snowballing was pursued to saturation, i.e., no new references appeared.

### 2.1. Review Topic

Assessment of the scientific evidence for clinical effects of TU in sport and companion animals.

### 2.2. General Inclusion and Exclusion Criteria

The inclusion criteria were that the publication is (a) in a peer-reviewed journal, (b) accessible through institutional access or internet search, and (c) a primary research publication. In the initial search stage, there were no restrictions with regards to either country or language of publication. The study should describe the physiological effects of TU or its efficacy in the treatment of a single indication in dogs, horses, or cats. The studies should be randomized controlled trials (RCTs), other interventional studies, or observational studies. A therapeutic intervention was defined as an intervention intended to reduce the signs, severity, or duration of a clinical condition. We also included experimental studies with induced lesions. Other laboratory experimental studies were included only if they mimicked a clinical situation and/or a mechanism of action was evaluated. Case series were included only if five or more subjects were included.

Because of the risk of confounding, we excluded intervention studies that involved any type of treatment concomitant to TU and not reporting a direct comparison with the concomitant intervention alone.

### 2.3. Study Selection and Categorization

In the screening phase, articles of possible relevance for the review were identified. The screening was based on journal title, publication title, or abstract. Citations identified were imported into Endnote (X9.3.3, 2018), and duplicates were removed. Two authors (ABo and HH) performed the selection and review of the articles.

Articles identified in the screening phase were selected for full-text evaluation. Articles not accessible from digital library resources were requested via the Swedish University of Agricultural Sciences Library.

For each study, the following key descriptive items were tabulated using templates modified from the Swedish Agency for Health Technology Assessment and Assessment of Social Services (SBU) [12]: first author, year of publication, study design, study population, intervention, control group, outcome, and relevance (external validity).

Assessment of the risk of bias (scientific quality) in each article was carried out in accordance with the Cochrane [13] and SBU guidelines [12]. The assessment was based on the following items: study design, statistical power, deviation from planned therapy, loss to follow-up, type of outcome assessment, and relevance. In observational studies, possible confounding was also included in the assessment. To ensure consistency, prior to starting the literature review, three of the authors (KA, HH, ABe) independently screened a random sample of articles; differences were discussed and resolved before reviewing all articles. Two of the authors (ABo and KA) first independently assessed the risk of bias of the papers included in the systematic review of TU, then discussed any discrepancies to reach a consensus. The certainty of evidence was assessed according to the GRADE system as very low, low, moderate, or high [14]. The writing of the paper has been conducted following the PRISMA 2022 checklist and the study has not been registered in PROSPERO since it is not for human health.

## 3. Results

### 3.1. Characteristics of the Literature

**Figure 1:**
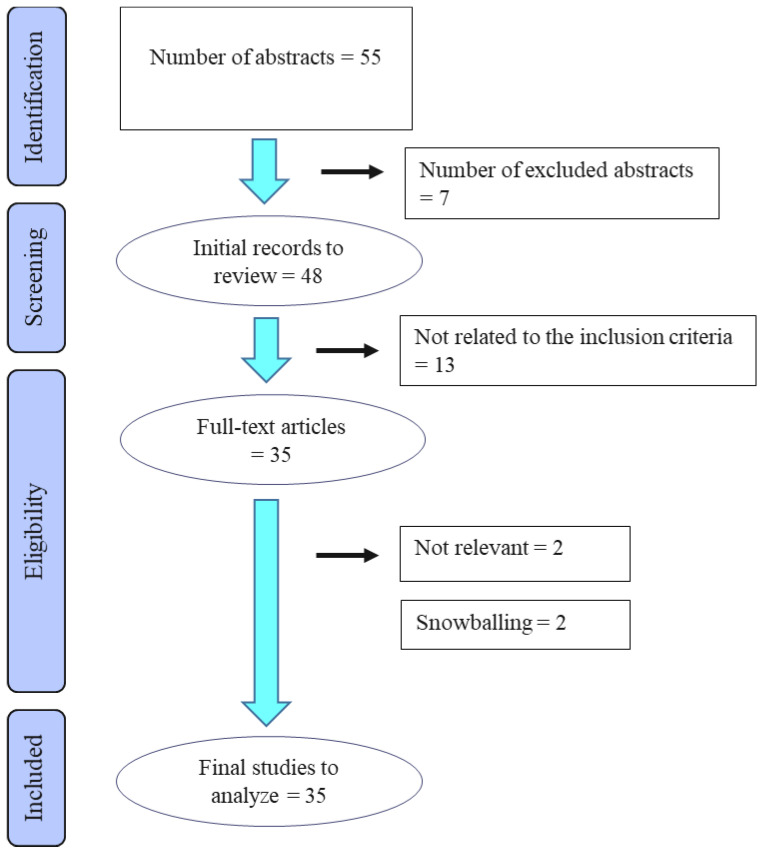
Flow diagram of the stages of the selection process used for identification of studies eligible for final analysis.

In the original literature search, a total of 33 articles on ultrasound treatment in animals met the inclusion criteria (see Figure 1). Further, snowballing retrieved two studies on donkeys, where both studies used the same population. Of these 35 included articles, 24 publications were on dogs, nine on horses and one on cats (two studies reported on several species). Twenty-one articles reported on experimental studies, three on two separate RCTs, four on non-randomized controlled studies, and seven on clinical observational studies without a control group. Although our literature search covered all possible indications, the articles retrieved were confined to the evaluation of TU either in musculoskeletal conditions or as a contraceptive intervention. At the evaluation of the risk of bias (study quality), 13 articles were assessed to have low, 12 moderate, and 10 high risk of bias.

**Figure 2:**
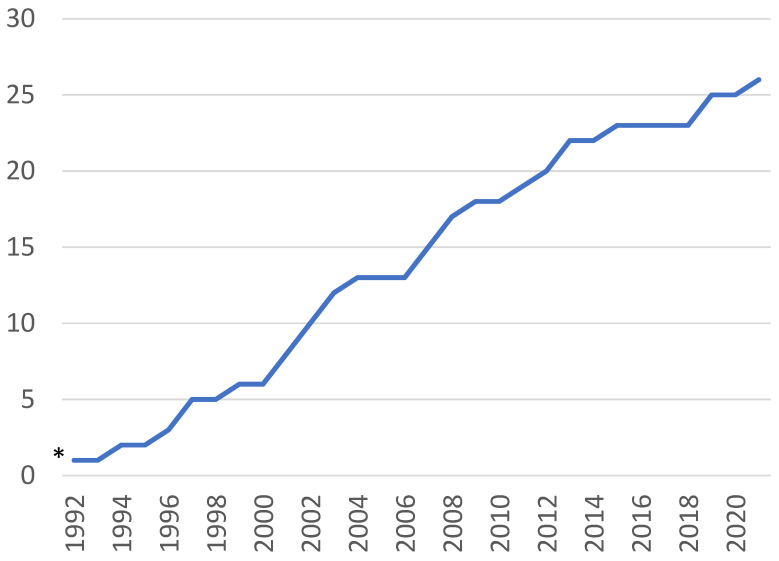
Cumulated number of 26 articles from the original literature search on the effects of TU in musculoskeletal conditions in sport and companion animals published during 1992–2021. * 1 article published in 1980.

The first article fulfilling our inclusion criteria was published in 1977, reporting on the effects of TU as a contraceptive intervention [15]. Apart from a US report in 1980 [16], the first articles on the use of TU for musculoskeletal conditions were published in India in the years 1994–1997 [17,18,19,20,21]. From 2001 onwards, there has been a steady increase in the number of articles published on TU for musculoskeletal disorders in dogs and horses, with a slowing down occurring after 2013, with only four articles being published during the years 2014–2020 (Figure 2). Two countries dominate the literature quantitively: India (11 articles) and the USA (11), with smaller numbers published from Brazil (three), Italy (three), Iran (two) and one each from Argentina, Japan, and Romania.

Key characteristics of the ultrasound treatment reported in the publications (number of sessions, treatment duration, mode (pulsed or continuous), frequency, and intensity (surface power density) are provided in Table 1 (musculoskeletal conditions in dogs), Table 2 (musculoskeletal conditions in horses and donkeys), and Table 3 (TU used for anticonception). The tables also summarize information on study populations, controls, outcome variables, main results, and each study’s risk of bias.

### 3.2. Musculoskeletal Conditions in Dogs

All the parameters for the subsequent papers are tabulated for review in Table 1.

#### 3.2.1. Tissue Temperature

A temperature rise induced by TU in the tissues of dogs was reported in four articles, all assessed to have a low risk of bias. Albuquerque et al. [22] reported on surface temperature changes, Steiss et al. [23] on deep muscle temperatures with an unclipped and clipped coat at different TU intensities, Levine et al. [24] on temperatures in muscles, and Acevedo et al. [25] on calcaneus tendon temperature and tarsal flexion. A total of 34 dogs were studied. Together, the results show that temperature rise induced by TU is higher at the surface than in deep tissues, higher with continuous mode than with pulsed mode, increases with elevated ultrasound intensity, and returns to baseline within 10 min after discontinuation (Table 1). When the coat is left unclipped, temperatures in deep muscle temperature rise very little [23]. In the study of Acevedo et al. [25], tarsal flexion was increased during treatment; flexion returned to baseline 5 min after the treatment was terminated.

*Overall assessment.* Experimental studies in sound dogs show consistent results on the effects of TU on tissue temperature. With continuous ultrasound at 1.5 W/mm^2^ intensity, the temperature in tendons rises by up to 3.5 °C, whereas the rise in muscles seems to be lower. The certainty of the evidence for these conclusions is assessed as moderate to high. The studies have not evaluated what temperature rise, if any, would be therapeutically optimal.

#### 3.2.2. Tendon Injury

One controlled experimental study on the healing of tendon injury was identified. The study was assessed to have a high risk of bias. Saini et al. [26] investigated healing in surgically severed Achilles tendons in three dogs treated by TU and compared this with healing in two non-treated animals [26]. The authors reported somewhat more rapid healing assessed clinically and by ultrasonography in TU-treated dogs over a 40-day follow-up compared to controls.

*Overall assessment.* The scientific support for use of TU to treat tendon injuries in dogs comes from a single small experimental study. The certainty of evidence is assessed as very low.

#### 3.2.3. Osteoarthritis

In a clinical cohort study without controls, Muste et al. [27] followed eight dogs diagnosed with osteoarthritis for up to one year after TU treatment. The authors reported improvement in clinical signs of osteoarthritis in all dogs.

*Overall assessment.* The scientific information on the effects of TU on osteoarthritis in dogs comes from a single cohort study without a control group and a high risk of bias. The certainty of evidence is very low.

#### 3.2.4. Other Limb Joint Lesions

In 1980, Lang [16] reported on 45 dogs with various limb joint lesions (including post-luxation symptoms, torn ligament, osteoarthritis, distortion, and synovitis); the lesions of 35 dogs were assessed as fully resolved at a non-specified time point when TU treatment was completed. There was no control group.

*Overall assessment.* The scientific information on the effects of TU on limb joint lesions of heterogeneous origin in dogs comes from a single cohort study with a very high risk of bias. The certainty of evidence is very low.

#### 3.2.5. Paraparesis, Hindquarter Weakness, and Disc Lesions

The clinical effects of TU in dogs with paresis, hindquarter weakness, and disc lesions have been studied in one RCT (reported in two articles) and three clinical cohort studies, one of them with and two without controls.

*Randomized controlled trial.* The results of an RCT involving 24 dogs with hindquarter weakness (HQW) have been reported in two separate articles [28,29]. The risk of bias was assessed to be moderate. Eight of the dogs were assigned to TU combined with conventional drug therapy and 8 received drug therapy only (the third group with eight dogs was treated with short-wave diathermy). All dogs were clinically improved by day 14. However, a somewhat higher proportion had normal hopping reactions at day 28 in the TU plus drug-treated group (4/8 dogs) than in the drug-only group (2/8 dogs). TU used in conjunction with conventional drug therapy seemed to counter HQW-associated oxidative stress in erythrocytes. The degree of improvement in oxidative stress in TU-treated dogs differed little from that of drug effects alone.

*Clinical cohort studies*. In their study of dogs with HQW, Maiti et al. [30] compared clinical effects in five dogs treated with TU and “conventional treatment” (unspecified) with five dogs receiving “conventional treatment” only. During the follow-up, pain was reported to be somewhat less severe in TU-treated dogs. The study had a high risk of bias.

When Sharma et al. [31] followed 16 dogs with different degrees of paresis treated by TU and concomitant medical treatment, 10 dogs were reported as fully recovered at an unspecified follow-up time. The patient population was highly heterogeneous, and there was no control group; the risk of bias was assessed as high.

In an observational study with a very high risk of bias, Lang [16] applied TU to 65 dogs with various types and locations of spinal lesions, mainly diagnosed as disc lesions. Based on clinical assessment at the end of treatment (unspecified time point), the study found that 48/65 dogs were fully recovered.

*Overall assessment.* The scientifically strongest study of TU to treat paralysis and HQW in dogs is a small RCT in which there were uncertain additional benefits of TU compared with conventional drug therapy alone. Results from clinical cohort studies with a high risk of bias also indicate that the effects of TU on paralysis and HQW are minor, if any. The dogs used in all of the above-mentioned studies had an unclear diagnosis or diagnostics were reported insufficiently. This contributed to the low certainty of the evidence for clinically important beneficial effects of TU in dogs with paralysis, HQW, or disc lesions.

#### 3.2.6. Bone Tissue and Bone Healing

In a safety study, Silveira et al. [32] investigated the effects of TU on healthy extremity bones of six dogs during 20 days of therapy. When assessed by radiography (with low sensitivity to detect small differences in bone density), TU did not affect bone density.

Seven studies on the effects of TU on bone healing in dogs were identified. They were all experimental, with surgically induced bone lesions, and in five of the studies, the risk of bias was assessed as low or moderate.

In an early randomized study, Singh et al. [17] compared bone histology at induced femoral fracture sites in TU-treated and non-treated dogs. By histomorphology, callus formation was assessed to be firmer and inflammatory reaction milder in the TU group, leading the authors to conclude that TU may enhance fracture healing. 

In another study with an identical methodology, the same authors’ induced humeral fractures in eight healthy dogs [21]. By histomorphology (unspecified time point), the authors observed less inflammatory reaction, better tissue differentiation, signs of improved callus strength, and less hyaline muscle degeneration in TU-treated dogs. These authors also used this same population to study haematological features and radiographical changes in TU-treated dogs [18]. The conclusion was that fracture healing was better in TU-treated dogs compared to controls [18].

Using a spine fusion model in 14 dogs, Cook et al. [33] studied bone healing after fusion surgery at two sites, one randomly allocated to TU for one week, the other receiving placebo treatment. At a 12-week follow-up, there was complete radiographic and histological fusion at all TU-treated sites. Of non-treated control sites, 78% had complete radiographic fusion and 44% had complete histological fusion. Mechanical stiffness was greater in the treated group at the 12-week follow-up. All differences were statistically significant.

Rawool et al. [34] observed by power Doppler sonography early signs of increased vascularity around the induced osteotomy area in TU-treated dogs compared with control dogs.

In a study without non-treated controls and therefore a high risk of bias regarding the effects of TU per se, Kaur et al. [35] observed earlier bone healing at radiography and earlier weight bearing at TU intensity 0.5 W/cm^2^ than at TU intensity 1.0 W/cm^2^ in dogs with induced radial diaphyseal defects.

Ikai et al. [36] evaluated how TU affected the healing of surgically induced bone defects in the mandibular bone. Defects were induced bilaterally with one side treated with and TU, the other serving as a control. At histological and immunohistochemical investigations 4 weeks after surgery, the authors observed accelerated regeneration of cementum and mandibular bone and higher expression of heat shock protein in gingival epithelial cells in the TU group.

*Overall assessment.* Although all published experimental studies have been small, we have, with a single exception [35], assessed them to have a low or moderate risk of bias. The scientific evidence is strengthened by the consistency of the reported results; thus, there is moderate certainty of evidence from experimental studies that TU promotes bone healing in dogs. There are no clinical trials of TU for non-experimental fractures in dogs.

**Table 1:** Characteristics of studies included in the systematic review of therapeutic ultrasound used to treat musculoskeletal conditions in dogs. TU, therapeutic ultrasound; US, ultrasound; HQW, hind quarter weakness; NI, no information.

Main Author [Ref.] Publication Year Country	Study Design	Study Population	Therapeutic Ultrasound: No. of Sessions and Duration Mode US Frequency Intensity	Controls	Outcome Variables	Main Results	Study Risk of Bias
Surface, muscle, and tendon temperature							
Steiss [23] 1999 USA	Experimental, before-after design	9 healthy dogs	One 10-min session Continuous mode TU frequency NI Intensity 0.5–2.0 W/cm^2^ Recordings with and without coat	Status before treatment	Temperature in biceps femoris recorded by thermistor	Temperature increase of >1.6 °C at 5 cm depth was obtained only when the coat was clipped and with intensity 2.0 W/cm^2^. No deep muscle temperature change with an unclipped coat	Low
Levine [24] 2001 USA	Experimental, treatments administered in random order	10 adult male and female dogs	One 10-min session Mode and TU frequency NI 1.0–1.5 W/cm^2^	Status before treatment	Caudal thigh muscle temperature at 1, 2, and 3 cm depth	1.0 MHz: temperature rise 3.0 °C at 1 cm, 1.6 °C at 3 cm. 1.5 MHz: temperature rise 4.6 °C at 1 cm, 2.4 °C at 3 cm	Low
Acevedo [25] 2019 USA	Experimental Prospective cross-over design without controls	10 adult dogs	One 10-min session Testing continuous vs. pulsed mode 3.3 MHz 1.0–1.5 W/cm^2^	Status before treatment	Calcaneus tendon temperature Tarsal flexion	Greatest increase in tendon temperature (mean 3.5 °C) with continuous TU at 1.5 W/cm^2^. Much smaller heating effect of pulsed TU (mean 1.5 °C). Tarsal flexion increased during treatment, returning to baseline within 5 min of discontinuation	Low
Albuquerque [22] 2021 Brazil	Experimental Before-after design	5 healthy adult dogs	One 10-min session Continuous mode 3.3 MHz 1.5 W/cm^2^	Status before treatment	Surface temperature monitored by thermography	Mean increase in surface temperature at end of the 10-min treatment was 3.8 °C, returning to baseline about 10 min after cessation of TU	Low
Tendon injury							
Saini [26] 2002 India	Experimental, non-randomized controls	5 healthy mongrel dogs with surgically severed Achilles tendons, all immobilized	Daily 10-min sessions for 10 days Mode NI TU frequency NI Intensity 1.5 W/cm^2^	Non-treated dogs	Follow-up for 120 days Clinical assessment Weight-bearing score Ultrasonography Tendon biopsy with histomorphology	Duration of lameness somewhat shorter in the TU group than in the controls. More rapid healing shown by ultrasonography and tendon histology	Moderate (only three treated and two non-treated dogs)
Osteoarthritis							
Muste [27] 2015 Romania	Clinical cohort without controls	8 dogs with hind limb knee osteoarthritis	10 daily sessions (with a 2-day break) Pulsed mode Frequency NI Intensity 0.5 W/cm^2^	None	10- to 12-month follow-up Degree of lameness Pain score Joint motion measured by goniometer	Improvement of joint mobility in all dogs, accompanied by reduced pain and lameness	High (no control, unclear and inconsistent information in the article)
Limb joint lesions							
Lang [16] 1980 USA	Clinical cohort without controls	45 dogs with various types of joint lesions	1–8 sessions, duration and intervals not specified. Pulsed mode Frequency NI Intensity 3 W/cm^2^	None	Clinical examinations with an assessment of swelling, pain, activity, and function at an unspecified time point. Radiography as needed	35/45 dogs’ symptoms fully resolved at end of treatment	High (heterogeneous cohort and TU treatments, follow-up not systematic, no controls)
Paraparesis, hindquarter weakness, and spinal lesions							
Lang [16] 1980 USA	Clinical cohort without controls	65 dogs with various types and locations of spinal lesions, mainly diagnosed as disc lesions	1–8 sessions, duration and intervals not specified Pulsed mode Frequency NI Intensity 3 W/cm^2^	None	Clinical examinations with an assessment of swelling, pain, activity and function at an unspecified time point.Radiography as needed	48/65 dogs’ symptoms fully resolved at end of treatment	High (heterogeneous cohort and TU treatments, follow-up not systematic, no controls)
Maiti [30] 2007 India	Clinical cohort, controlled, non-randomized	15 adult dogs with hindquarter weakness	Three 10-min sessions per week until discharge Pulsed mode 1 MHz 2 W/cm^2^	Conventional therapy and US (n = 5), conventional therapy and interferential therapy (n = 5), conventional therapy only (n = 5)	Clinical neurological examination with the grading of motor function on days 3, 7, 10, and 14 and thereafter once a week. Biochemical measurements Radiographs of the spine	Less pain in TU in comparison with the control group receiving only conventional therapy	High (small sample, insufficient details on clinical assessments)
Sharma [31] 2011 India	Clinical cohort without controls	16 dogs with different grades of paraparesis, all receiving medical treatment ambulatory or non-ambulatory	5- to 10-min sessions twice weekly until discharge Pulsed mode Frequency NI Intensity 1.5–2.0 W/cm^2^	None	Clinical, neurological examination, restoration of activity and function. Blood samples, heart rate, respiratory rate, rectal temperature. Duration of follow-up not specified.	Symptoms fully resolved in 10/16 dogs after 2–10 treatment sessions	High (heterogeneous sample, no controls)
Ansari [28] 2012 Zama [29] 2013 India	Randomized controlled trial	16 dogs diagnosed with hindquarter weakness (HQW) 8 healthy age-matched control dogs	Daily 5-min sessions for 14 days Pulsed mode 1.0 MHz 0.5 W/cm^2^	Conventional drug therapy	Clinical assessment Postural reactions Erythrocyte oxidant-antioxidant balance followed for 28 days by lipid peroxidation, reduced glutathione, superoxide dismutase and catalase	All dogs had improved postural reactions, except for the hopping reaction, by day 14. At day 28, hopping was improved in 4/8 dogs in the TU group vs. 2/8 in the control group. Clinical improvement was reported (without details) in all dogs in both groups. TU with conventional drug therapy seemed to counter oxidative stress associated with HQW. The degree of improvement differed little from drug effects alone	Moderate
Bone tissue and bone healing							
Silveira [32] 2008 Brazil	Clinical cohort, controlled	6 healthy dogs	TU to distal-skull area of radius and ulna Daily 5-min sessions for 20 days Continuous mode 1.0 MHz 0.5 W/cm^2^	Non-treated contralateral radius and ulna	Bone density by radiography	No effects of TU on bone density at 20-day follow-up	Moderate (lower US dosage than in most other studies)
Singh [17] 1994 India	Experimental, randomized controlled trial	8 healthy mongrel dogs with experimental femoral fracture	Ten 5-min sessions, alternate days, Pulsed mode Frequency NA Intensity 0.5 W/cm^2^	Non-treated dogs	Histomorphology, calcium deposits, muscle fibre striation after euthanasia at day 40 postoperatively	Callus formation was firmer, inflammatory reactions milder in the TU group than in controls.	Moderate (small number of animals, no quantitative data provided)
Singh [18] 1994 Singh [21] 1997 India	Experimental, randomized controlled trial	8 healthy mongrel dogs with experimental humerus fracture	Ten 5-min sessions, alternate days, Mode NI Frequency NI Intensity 1.0 W/cm^2^	Non-treated dogs	Histomorphology at an unspecified time point. Clinical observations, angiography, radiography	Less inflammatory reaction, better tissue differentiation, signs of better callus strength and less hyaline muscle degeneration in TU-treated dogs. Faster healing detected in radiographs in TU-treated dogs.	Moderate (small number of animals, no quantitative data provided)
Cook [33] 2001 USA	Experimental Canine spinal fusion model, controlled	14 adult male dogs with posterior spinal fusions at two sites (L2–L3 and L5–L6)	6–7 daily 20-min sessions for 6 or 12 weeks Pulsed mode 1.5 MHz 30 mW/cm^2^	Placebo-treated spinal fusion site in the same dog	Evaluation at 6 and 12 weeks Palpation Torsional stiffness Radiographic grading CT and MRI Histology	Complete radiographic and histological fusion at 12 weeks in 100% of sites receiving TU. Of non-treated control sites, 78% had complete radiographic fusion and 44% had complete histological fusion. Mechanical stiffness improved in the treated group. Differences were statistically significant.	Low
Rawool [34] 2003 USA	Experimental, controlled	6 dogs with ulnar osteotomies	One 20-min session Pulsed mode 1.5 MHz 30 mW/cm^2^	Placebo-treated dogs (device turned off)	Vascularity around osteotomy measured by power Doppler sonography for 11 days	Increased vascularity in TU-treated dogs at 7 and 11 days compared with placebo-treated dogs	Moderate (small number of TU-treated and control animals)
Kaur [35] 2004 India	Experimental, controlled	10 adult healthy dogs with experimental radial diaphyseal defect	Daily 10-min sessions for 10 days Pulsed mode US frequency NI Comparison of 0.5 and 1.0 W/cm^2^	None	Clinical examinations, radiography, and angiography before and up to 60 days after surgery	Earlier bone healing and weight bearing at 0.5 W/cm^2^ compared with 1.0 W/cm^2^	High (no non-treatment controls)
Ikai [36] 2008 Japan	Experimental, controlled	4 healthy beagle dogs with surgically induced bone defects in the mandibular bone bilaterally	Daily 20-min sessions for 4 weeks. Pulsed mode Burst 1.5 MHz, repeated treatments at 1.0 kHz 30 mW/mm^2^	Non-treated contralateral side	Histology and immunohistochemistry 4 weeks after surgery	Accelerated regeneration of cementum and mandibular bone in the TU group. Expression of heat shock protein higher in gingival epithelial cells of the US-treated tooth	Low

### 3.3. Musculoskeletal Conditions in Horses and Donkeys

All the parameters for the subsequent papers are tabulated for review in Table 2.

#### 3.3.1. Tissue Temperature

We identified two experimental studies in which temperature changes induced by TU were investigated in horses: one in muscles, the other in tendons. In a study with a low risk of bias, Adair et al. [37] measured temperature changes in the epaxial muscles of 10 healthy horses. During ultrasound treatment with an assessment of therapeutic frequency and intensity, muscle temperature increased by 1.2–2.5 °C, being highest at depths of 1 and 5 cm and lowest at 3 cm. After the end of treatment, the temperatures gradually returned to baseline levels but were still somewhat elevated (by 0.4–1.1 °C) 10 min after termination of treatment.

In a similar experimental study, assessed to have a low risk of bias, Montgomery [38] measured temperature changes in the tendons and muscles of 10 healthy horses. At the end of a 10- to 20-min treatment session, the temperature had increased by 2.5–5.2 °C in superficial and deep flexor tendons of the thoracic limb. In epaxial muscles, the temperature rise was much less (0.7–1.3 °C), deemed by the authors as a non-therapeutic temperature rises.

*Overall assessment.* Two experimental studies in sound horses showed temperature rises in muscles during treatment. At the same ultrasound frequency and intensity, the temperature rise seems to be considerably higher in tendons. The certainty of this evidence is assessed as moderate to high. The studies have not evaluated which temperature rise, if any, would be therapeutically optimal.

#### 3.3.2. Tendon Injury and Inflammation

Two experimental studies with induced tendon injuries and two clinical cohort studies without controls were identified. In an RCT with a low risk of bias, tendon injury was induced by collagen injections in 18 horses [39]. The horses were randomly allocated to treatment with continuous mode TU or pulsed mode TU or to a control group. TU-treated horses in both groups showed earlier clinical improvement and less severe tendinitis by ultrasound than control horses at 40 days’ follow-up. At histological examination, TU-treated horses had more intense neovascularization and fibroblastic activity than controls.

In a study with a moderate risk of bias, four horses with experimentally induced lacerations of the superficial digital flexor tendon were treated with TU, with four untreated horses serving as controls [40]. The authors reported local tenderness and swelling to be less severe in the TU-treated limbs during the 63-day follow-up. After laceration of the tendon, the tendon content of hydroxyproline decreased; the decline was less in the treated limb, which according to the authors indicated improved tendon regeneration.

In a clinical cohort study without controls, Carrozzo et al. [41] used low-frequency ultrasound to treat 23 sport horses with desmitis for 3–4 weeks. By diagnostic ultrasound, all but three horses showed healing and were reported to have returned to “full competition status” within 3–8 months. However, there was no control group and the study was rated to have a high risk of bias.

In a study with a very high risk of bias, Lang [16] reported on the outcome of 10 horses with synovitis and bursitis treated with TU [16]. There were varying treatment parameters, no fixed follow-up time, and no controls. The author assessed five of the horses as fully recovered at the end of treatment.

*Overall assessment.* The certainty of the evidence for the beneficial effects of TU in horses with tendon injury or inflammation is low to moderate.

#### 3.3.3. Acute Aseptic Arthritis

In two publications, the results of an RCT in eight donkeys with acute septic arthritis were reported by Singh et al. [19,20]. Both reports were assessed to have a moderate risk of bias. The authors observed earlier improvement of lameness, faster reduction of joint swelling, and less pain on flexion in the four donkeys assigned to TU than in the four non-treated donkeys. Synovial cytological and biochemical analyses indicated less inflammation in the TU group, and at histomorphological examination, there were signs of improved healing and fewer signs of cartilage degeneration in TU-treated donkeys.

*Overall assessment.* The evidence for the beneficial effects of TU in donkeys with acute septic arthritis or inflammation is therefore assessed as low. In horses, no studies are available.

#### 3.3.4. Back Muscle Pain

In a controlled clinical cohort study of 63 show-jumping horses with back pain, the effects of TU alone were compared with TENS and the combination of the two therapies [42]. Because of the lack of an untreated control group, the risk of bias was assessed as high for addressing the question of TU effects alone. The authors found that TU alone was associated with more rapid recovery than TENS alone at 63 days’ follow-up and that there was a supplementary effect of adding TENS to TU.

*Overall assessment.* A single study has reported positive results of TU in horses with back muscle pain, but the study lacked an untreated control group. The certainty of the evidence for the beneficial effects of TU in horses with back muscle pain is therefore assessed as low.

#### 3.3.5. Spinal and Other Bone and Joint Lesions

In a mixed group of 30 horses in which spinal lesions and other bone and joint lesions had been diagnosed, Lang [16] reported that 24 of the horses recovered after an unspecified length of time following TU treatment.

*Overall assessment.* The certainty of the evidence for the beneficial effects of TU in horses with spinal lesions and other bone and joint lesions is very low.

**Table 2:** Characteristics of studies included in the systematic review on therapeutic ultrasound used for musculoskeletal conditions in horses and donkeys. TU, therapeutic ultrasound; US, ultrasound; HQW, hind quarter weakness; NI, no information.

Main Author [Ref.] Publication Year Country	Study Design	Study Population	Therapeutic Ultrasound: No. of Sessions and Duration Mode TU Frequency Intensity	Controls	Outcome Variables	Main Results	Study Risk of Bias
Muscle and tendon temperature							
Adair [37] 2019 USA	Experimental, before-after design	10 healthy mares	One 10-min session Mode NI 1 MHz 1–2 W/cm^2^	Baseline temperature	Temperature measured by a thermistor at different depths in epaxial muscles	Muscle temperature rise at the end of treatment 1.2–2.5 °C, highest at 1 and 5 cm depth	Low
Montgomery [38] 2011 USA	Experimental, before-after design	10 healthy horses	Tendons: One 10-min session Continuous mode 3.3 MHz Comparing 1.0 with 1.5 W/cm^2^ Epaxial muscles: One 20-min session Continuous mode 3.3 MHz 1.5 W/cm^2^	Baseline temperature	Temperature monitored by a thermistor in the superficial and deep flexor tendons of the thoracic limb and in epaxial muscles	The temperature rises at the end of treatment 2.5–5.2 °C in tendons (therapeutic temperature rise according to authors) and 0.7–1.3 °C in epaxial muscles (non-therapeutic temperature rise)	Low
Tendon and ligament injury and bursitis							
Lang [16] 1980 USA	Clinical cohort without controls	10 horses with synovitis and bursitis	1–8 sessions, duration and intervals not specified. Pulsed mode TU frequency NI Intensity 3 W/cm^2^	None	Clinical examinations with an assessment of swelling, pain, activity, and function at an unspecified time point. Radiography as needed	5/10 horses fully resolved at end of treatment	High (heterogeneous cohort, follow-up not systematic)
Fernandes [39] 2003 Brazil	Experimental, randomized controlled trial	18 horses with collagen-induced injuries of superficial digital flexor tendon	8 sessions 2 intervention groups, one with continuous and one with pulsed mode 3 MHz 1 W/cm^2^	Device switched off	40 days’ follow-up Clinical examinations UltrasonographyHistology	Regression of clinical signs was detected on average in 9 days in horses receiving continuous US, 12 days in horses receiving pulsed mode US, and 21 days in controls. Decreases in clinical severity by ultrasonography after 40 days were 42.5%, 57.7%, and 34.1% in the three groups, respectively. Intense neovascularization and fibroblastic activity in US-treated groups compared with controls	Low
Sharifi [40] 2007 Iran	Experimental, non-randomized controls	8 castrated horses with experimentally induced lacerations of the superficial digital flexor tendon of the right hind limb	Daily 10-min sessions for 14 days 3 MHz Intensity 1 W/Esq	Non-treated horses	Clinical assessment and hydroxyproline content in the lacerated tendon at 60 days	Local tenderness and swelling were reported as less severe in treated limbs. Significantly less decline in tendon hydroxyproline tendon content in the treated limb, indicating improved tendon regeneration	Moderate
Carrozzo [41] 2019 Italy	Clinical cohort without controls	23 client-owned sport horses with injuries to the suspensory ligament	6 sessions 1st week, thereafter 3–6 sessions per week for 2–3 weeks 6 min 38 kHz Intensity NI	None	Clinical evaluation Time to healing by diagnostic ultrasound Return to competition status	20/23 horses showed healing by diagnostic ultrasound and returned to competition status	High
Acute aseptic arthritis							
Singh [19] 1996 Singh [20] 1997 India	Experimental, randomized controlled trial	8 donkeys with aseptic arthritis induced in the left carpal joint	Daily 10-min sessions for 7 days Pulsed mode 1 MHz 1.0–1.5 W/cm^2^	Untreated donkeys	30-day follow-up Rectal temperature, respiratory rate, pulse rate, and joint circumference. Synovial biopsies. Clinical assessments Histopathology and histochemistry	Earlier improvement of lameness, faster reduction of joint swelling, and less pain on flexion in TU-treated donkeys than in non-treated donkeys. Synovial cytological and biochemical parameters indicate less inflammation in the TU group. Histomorphology: Improved healing and fewer signs of cartilage degeneration in TU-treated donkeys	Moderate (only four donkeys in each group)
Back muscle pain [myositis]							
Mercado [42] 2002 Argentina	Clinical cohort, non-randomized controls	63 showjumpers with back pain (longissimus dorsi muscle)	Daily 20-min sessions for 30 days Pulsed mode TU frequency NI 3.5 W/cm^2^	TU alone compared with (a) TENS and (b) TU plus TENS	Weekly clinical assessments and ultrasonography, 28-day follow-up	Nearly total recovery at 28 days in the TU alone group, faster than with TENS treatment alone, but slower than in the group with TU combined with TENS	Moderate (no non-treated controls)
Spinal and other bone and joint lesions							
Lang [16] 1980 USA	Clinical cohort without controls	30 horses with various types and locations of spinal and other bone and joint lesions	1–8 sessions, duration and intervals not specified. Pulsed mode TU frequency NI 3 W/cm^2^	None	Clinical examinations with an assessment of swelling, pain, activity, and function at an unspecified time point. Radiography as needed	24/30 horses fully resolved at end of treatment (unspecified time point)	High (heterogeneous cohort, follow-up not systematic, no controls)

### 3.4. TU for Contraception in Dogs and Cats

We identified four articles in which contraceptive effects of TU were evaluated; of these, three had studied dogs exclusively and one both dogs and cats (all the parameters for the subsequent papers are tabulated for review in Table 3).

In a study with a low risk of bias, Fahim et al. [15] reported the results of 2–3 TU sessions in inhibiting spermatogenesis in 30 dogs and 30 cats and suggested a potential contraceptive use. Blood testosterone levels were unaffected.

In a study with a low risk of bias, Leoci et al. [43] treated five dogs with three sessions of ultrasound of the testicles. Fourteen days after the TU sessions, the authors observed testicular tenderness, reduced testicular size, and azoospermia.

The same research group performed an RCT of TU for contraception. Altogether 100 dogs were randomized to one of four intervention groups with different TU regimes and one control group [44]. The study was assessed to have a low risk of bias. In the group receiving three sessions of TU at 48-h intervals, a 30-day follow-up showed reduced testicular size and azoospermia.

Khanbazi et al. [45] performed an experimental RCT of TU involving 10 dogs (moderate risk of bias). At follow-up 63 days after the start of TU to the testicles, there was no difference in semen quality or testosterone levels between actively treated and control dogs.

*Overall assessment.* With the caveat that the results reported were not entirely consistent, the certainty of the evidence for repeated sessions of TU at high intensity to induce azoospermia and testicular degenerative changes in dogs and cats was assessed as moderate to high. There are no long-term studies on whether the TU effects are temporary or permanent.

**Table 3:** Characteristics of studies included in the systematic review of therapeutic ultrasound used in contraception. TU therapeutic ultrasound, NI no information.

Main Author [Ref.] Publication Year Country	Study Design	Study Population	Therapeutic Ultrasound: No. of Sessions and Duration Mode TU Frequency Intensity	Controls	Outcome Variables	Main Results	Study Risk of Bias
Dogs							
Fahim [15] 1977 USA	Experimental intervention study, before-after design	Experiment 1: 24 adult healthy dogs Experiment 2: 6 adult healthy dogs	Experiment 1: 1-3 15-min sessions, weekly intervals Mode NI TU frequency NI Intensity 1 W/cm^2^ Experiment 2: One 15-min session Intensity 2 W/cm^2^	None (comparisons between different ultrasound treatments)	Follow-up at 60 days Semen analyses (sperm count) Blood testosterone	Zero sperms after 60 days with three treatments at 1 W/cm^2^, 70% reduction with two treatments, 40% with one treatment. After one treatment with 2 W/cm^2^, spermatogenesis ceased after 14 days. Sperm stem cells and Sertoli cells were not affected. No changes in blood testosterone levels	Low
Leoci [43] 2009 Italy	Experimental intervention study, before-after design	5 healthy mixed-breed male dogs	Three 5-min sessions with 2-day intervals Mode NI US frequency NI Intensity 1.5 W/cm^2^	Status before TU	14-day follow-up Testicular size and tenderness Semen analysis (sperm count)	Testicular tenderness and reduced testicular volume. All dogs azoospermic at 14 days’ post-treatment	Low
Leoci [44] 2015 Italy	Randomized controlled trial	100 mature healthy mixed-breed maledogs	Five intervention groups: One to three 5-min sessions with 5-min to 48-h intervals and different areas of testicles treated Mode and frequency NA Intensity 1.5 W/cm^2^	Non-treated (device switched off)	On day 30: Testicular size, sperm evaluation, blood testosterone.At days 40–47: Histological evaluation of the testicles	Reduced testicular size, azoospermia, and testicular tissue degeneration in dogs receiving three sessions of TU over the entire testicles at 48-h intervals, 1.5 W/cm^2^. No major effects of other regimens. No changes in testosterone levels in any experimental group	Low
Khanbazi [45] 2020 Iran	Experimental randomized study	10 mixed-breed adult fertile dogs	Three 5-min sessions, thereafter one session every 48 h (NI on for how long) 1.0 MHz 1.5 W/cm^2^	Placebo-treated (device turned off)	63-day follow-up Ultrasound of testis Semen evaluation Serum testosterone and other biomarkers Oxidative stress index Histology	No difference in semen quality or testosterone levels Temporary increases in inflammatory and oxidative stress biomarkers Histological signs of late testicular necrosis and degeneration	Moderate (some information on the TU regime lacking)
Cats							
Fahim [15] 1977 USA	Intervention study, before-after design	30 male cats	Group A: One 10-min session Mode NI US frequency NI Intensity 1 W/cm^2^ Group B: same plus repeated same treatment at 48 h	Untreated cats	Follow-up at 60 days Histology in testicular biopsies Blood testosterone	Suppression of spermatogenesis at 60 days, more pronounced after two treatments. Unchanged testosterone levels	Low

## 4. Discussion

Our systematic review found that, in addition to experimental mechanistic studies, evaluations of therapeutic ultrasound (TU) effects on tendon and bone healing, osteoarthritis, paraparesis, and hindquarter weakness in dogs have been published. In horses, TU effects have been investigated in tendon and ligament injuries, acute aseptic arthritis, and back muscle pain. The effects on spermatogenesis have been studied in both dogs and cats. With two possible exceptions, the certainty of scientific evidence support has been assessed to be low or very low. As we discuss below, the exceptions concern bone healing and contraceptive effects.

Of the 26 publications reviewed on TU effects on musculoskeletal function, nine met the criteria for low risk of bias. Of all articles, more than half were experimental studies, either short-term laboratory experiments or induced injuries in otherwise healthy animals; most of these articles had a low risk of bias. There were, however, only occasional clinical studies with a low risk of bias. Several of the clinical studies were observational without a control group (or an inadequate control group). This makes it difficult to evaluate the extent to which reported favourable effects of TU could be discriminated from spontaneous recovery. When a control group had been included, few of the studies were large enough to have sufficient statistical power to detect clinically meaningful differences between the groups. With many small studies, the risk of a type I error (a false-positive finding) in any of them increases.

The reasons why only a few high-quality studies of TU have been performed in sport and companion animals may be diverse. With an owner-driven market for a method such as TU, the incentives to improve the scientific evidence may be limited; presumably, few customers ask for scientific documentation. In addition, many complementary and alternative veterinary medicine (CAVM) therapists may be sceptical of the design of academic studies. In the human complementary and alternative medicine community, the reliance on randomized controlled studies as the golden standard to evaluate the effects of therapy has been criticized [46]. The study design of some of the reports covered by our review also indicates that there is limited experience in how to conduct research of sufficient quality to convince the research and veterinary practice communities to accept (or discard) the therapy.

In contrast to many other CAVM therapies, TU is based on conventional explanatory models generally accepted by the scientific community. In the present review, we identified four studies in dogs and two studies in horses that confirmed heating effects in muscles and tendons. However, due to heterogeneity in parameter selection to enable comparisons between studies- there is uncertainty about the optimal temperature rise to achieve clinically meaningful results without causing adverse effects.

With the exception of intended testicular tissue degeneration when TU is used for contraceptive purposes, no major long-term adverse effects of TU have been reported in the articles included in the present review. Although no adverse effects of TU have been reported, the possible contraceptive effects of TU suggest that TU may be contraindicated for use in musculoskeletal disorders in areas adjacent to the testicles (groin area or proximal parts of medial thigh muscles).

For most of the neuromuscular indications, there was insufficient scientific evidence for favourable clinical effects of TU; the studies have been (a) negative or (b) of insufficient quality or (c) the results have been contradictory or (d) confirmatory results are lacking. For bone healing, however, several experimental studies with a low risk of bias have reported consistent results with improved early healing after induced bone fracturing or other surgical bone lesions. The experimental evidence seems sufficiently strong to initiate studies of the effects of TU in well-designed clinical trials of TU to treat non-induced bone fractures in clinical canine or equine patients. This notion is supported by results in humans with fractures. A recent literature review by Palanisamy et al. [47] concluded that favourable effects on the healing time of fresh fractures and the healing of non-unions have been documented.

As this review shows, some studies on the use of TU for contraception in dogs and cats have been published. In these studies, the optimal intensity of treatment to abolish spermatogenesis has been reasonably well-defined. In none of the studies was the follow-up extended beyond 60 days. Long-term studies appear to be needed to explore whether azoospermia is transient or persistent and to monitor possible late adverse effects of TU on contraception.

### Strengths

This is the first systematic literature review of studies on the effects of TU in sport and companion animals. Established methods for literature search, selection of relevant articles, and abstracting of information from the articles were used. As in the other articles on CAVM methods used in sport and companion animals reviewed in this issue of *Animals*, assessments of risk of bias in individual articles were based on templates developed by the Cochrane Collaboration [13] and the Swedish Agency for Health Technology Assessment and Assessment of Social Services (SBU) [12]. In addition, the certainty of the evidence was assessed by the well-established GRADE system [14] and professional librarians performed the literature search.

The broad search strategy had low specificity, with a small proportion of articles initially identified by title and/or abstract eventually fulfilling the inclusion criteria. On the other hand, sensitivity was reasonably high. Yet, a number of articles were identified using snowballing. To mitigate the problem of possible non-identified relevant articles, snowballing was pursued until maturation, i.e., no more relevant articles were retrieved.

A major obstacle to drawing conclusions on treatment effects of TU in sport and companion animals is the marked heterogeneity of the studies included in the review; this concerns study design, selection of treatment parameters and protocols, use of controls, statistical power, outcome measurements, and follow-up time. The many low-quality publications among clinical studies reduce the possibility of drawing conclusions but bear in themselves witness to the insufficient scientific evidence for many of the methods.

Considering the heterogeneity between studies and the low number of studies for each combination of species and indication, pooled statistical analysis with meta-analysis was not feasible. With many small studies, the possibility of publication bias increases; small studies with a negative outcome are less likely to be published [48]. The methods to detect and adjust for publication bias require that there are at least some larger studies to be used as a reference [49]. For most methods covered by the present systematic review, the number of publications was small and the intervention groups consisted of 10 animals or less. Construction of funnel plots or the use of other methods to detect possible publication bias was therefore not feasible.

As the aim of the systematic literature review was to evaluate the scientific literature on sport and companion animals, the initial search was restricted to dogs, horses, and cats. Further, through snowballing also donkeys were included because of their similarities to horses. Had more species been included, additional publications could possibly have been retrieved. Considering potential between-species differences in pathologies, extrapolation of results from one species to another is, however, problematic.

## 5. Conclusions

This systematic review revealed significant gaps in scientific knowledge regarding the clinical effects of TU in dogs, horses, and donkeys. For TU in cats, only one article (on TU for contraception) was retrieved. Most published articles concern musculoskeletal conditions. From several experimental articles with a low risk of bias, there is moderate scientific evidence that TU promotes bone healing, at least in dogs; these findings require confirmation in high-quality clinical trials. There is also moderate scientific evidence that TU induces azoospermia in dogs and cats, an effect that may be used in contraception.

Our review has shown that there is moderate scientific evidence that ultrasound administered with certain dosing can cause a heat increase in muscles and tendons in healthy animals. These heat increases and possible non-thermal effects of ultrasound could tentatively be of benefit in the treatment of clinical pain conditions or injuries. However, in injuries and diseases affecting muscles, tendons, and ligaments, there is insufficient scientific evidence for the clinical effects of TU. In the published clinical studies, the numbers of animals studied are small, control groups are often lacking, and there are other methodological limitations. Thus, only a few clinical articles with a low or moderate risk of bias were identified. When favourable results were reported, they were seldom replicated in independent studies. The large proportion of studies with a high risk of bias emphasizes the need for more high-quality research using well-established research methodologies to evaluate the potential therapeutic effects of TU in sport and companion animals.

## Data Availability

Not applicable.
